# Efficacy of perampanel in pediatric epilepsy with known and presumed genetic etiology

**DOI:** 10.1002/acn3.51828

**Published:** 2023-06-16

**Authors:** Pu Miao, Xueying Zhu, Wenqin Jin, Lingyan Yu, Yanfang Li, Ye Wang, Qunyan Su, Sha Xu, Shuang Wang, Jianhua Feng

**Affiliations:** ^1^ Department of Pediatrics Second Affiliated Hospital, School of Medicine, Zhejiang University Hangzhou 310009 China; ^2^ Department of Pediatrics Jinhua Lanxi People's Hospital Jinhua Zhejiang 321000 China; ^3^ Department of Pharmacy Second Affiliated Hospital, Zhejiang University School of Medicine Hangzhou China; ^4^ Department of Pediatrics Taizhou Woman and Children's Hospital Taizhou 318000 China; ^5^ Department of Neurology, Epilepsy Center Second Affiliated Hospital, School of Medicine, Zhejiang University Hangzhou 310009 China

## Abstract

**Objective:**

The efficacy of perampanel (PER) in pediatric epilepsy with specific etiologies has not been well established. Here, we investigated outcome and predictors of PER treatment in a pediatric cohort with known and presumed genetic etiology.

**Methods:**

We included pediatric patients with potential genetic epilepsy who received PER treatment and underwent whole‐exome sequencing (WES) from January 2020 to September 2021. All patients were followed up for >12 months.

**Results:**

A total of 124 patients were included. Overall response rates were 51.6% and 49.6% at 6 months and 12 months, respectively. Pathogenic or likely pathogenic variants in 27 multiple genes were detected among 58 patients (46.8%) by WES. On performing multivariate logistic regression analysis, only developmental delay (OR = 0.406, *P* = 0.042) was a negative predictor of treatment response. However, the seizure onset age, positive WES results, and number of ASMs before PER administration were not significantly. Thirteen carriers with variants in the *SCN1A* gene showed a better response compared to eight patients with other sodium channels (*P* = 0.007), and to the other 45 patients with positive WES results (OR = 7.124, 95% CI = 1.306–38.860, *P* = 0.023). Adverse events were only reported in 23 patients, the most common being emotional problems.

**Interpretation:**

PER is safe and efficacious in pediatric patients with known and presumed genetic etiology. The response rate is comparable to that reported in other pediatric populations, and lower among those with developmental delay. A gene‐specific response to PER is found along with better efficacy links to pathogenic variants in the *SCN1A* gene.

## Introduction

Epilepsy is a common chronic neurologic disorder in children. About one out of 150 children are diagnosed with epilepsy during the first 10 years of their life.^1^ The etiology of epilepsy is a major determinant of medical treatment response. Underlying etiologies of pediatric epilepsy are heterogeneous, of which genetic or presumed genetic sources occupy one‐third of childhood‐onset epilepsy^2^, ^3^ and up to 80% of infant‐onset epilepsy.^4^ Genetic detection has recognized several forms of genetic epileptic encephalopathy. Among them, Dravet syndrome is commonly caused by *SCN1A* pathogenic variants and presents with drug‐resistance and neurodevelopmental comorbidities.^5^ Advancements in genetics have provided essential information on the precise treatment approaches to epilepsy. In patients with gain of functional variants in *SCN8A* or pathogenic variants in the *KCNQ2* gene, traditional sodium channel blockers can reduce seizures.^6^ Sirolimus, an mTOR inhibitor, is precision therapy for epileptic patients with tuberous sclerosis.^7^ Nevertheless, not all patients can get a definitive genetic diagnosis and not all patients with definitive genetic etiology can obtain seizure reduction with anti‐seizure medications (ASMs). Novel effective treatments are important for pediatric patients with epilepsy not only because childhood is a crucial time for brain development, but also because uncontrolled epilepsy has much higher all‐cause morbidity and mortality rates than epilepsy in remission.

Perampanel (PER) is a selective noncompetitive AMPA‐receptor antagonist approved for the treatment of focal onset seizures for patients ≥4 years of age and primary generalized tonic–clonic seizures for patients ≥12 years of age. Several studies demonstrated PER is efficient and safe in patients under 4 years of age with epilepsy.^8^, ^9^, ^10^ Most of these studies were based on drug‐resistant epilepsy with mixed etiologies. There are several anecdotal reports describing successful usage of PER in epileptic patients with definite genetic etiology.^11^, ^12^ Qu et al. recently reported that PER, as an adjunctive treatment, effectively reduced seizures in 46% of the cohort consisting of 50 children with drug‐resistant genetic epilepsy.^13^ However, the treatment response of PER in the general population of genetic etiology is not well known. Additionally, whether there is a gene‐specific treatment response for PER, requires further investigation.

Our study aims to investigate the efficacy and safety of PER in a cohort of pediatric epilepsy with known and presumed genetic etiology. The treatment outcome and its predictors were evaluated after following up for >12 months. The impact of gene‐special etiology on treatment response was also examined for those with definite genetic etiology revealed by the whole‐exome sequencing (WES).

## Methods

### Patients

This observational, retrospective study was conducted in the second affiliated hospital of Zhejiang university. It was approved by the ethics committee in our hospital (2021‐0605/IR2021398). We extracted clinical characteristics from the medical database of pediatric patients (<18 years of age) with focal or generalized epilepsy who received PER treatment during January, 2020–September, 2021. Patients with insufficient clinical data or who were absent for follow‐up were excluded.

Epilepsy with potential genetic etiology was defined according to the International League Against Epilepsy (2017). Inclusion criteria for patients were as follows: (1) neonatal or infantile onset epilepsy (exclude acquired etiologies); (2) have a family history of epilepsy (≥1 first degree relative or ≥2 second degree relatives); (3) unknown causes of developmental and epileptic encephalopathy by routine inspection; (4) combination with developmental delay or autistic phenotype, especially before seizure onset; (5) combination with apparent deformity, growth retardation, or feeding difficulties; (6) combination with cortical malformations, which is evaluated by magnetic resonance imaging (MRI) scan with epilepsy protocol; (7) accordant with particular phenotypes (such as tuberous sclerosis and Dravet). Acquired epilepsy as the etiology of immune, infection, stroke, tumor, trauma, and any other un‐assumed genetic reason were excluded. These patients' sample, with potential genetic etiology, was used for WES.

Data retrieved from the electronic database were gender, age, age at onset of seizure, seizure features (including being fever‐sensitive or not, seizure types), number of all ASMs taken so far (including current medication), head MRI, electroencephalogram, and results of WES sequencing according to ACMG guidelines. Initial doses of PER were individualized according to patients' age, weight, and clinical condition: at 2 mg once daily (patients ≥4 years and weights ≥30 kg); at 1 mg once daily (patients ≥4 years and weights ≥20 kg, < 30 kg); and at 0.5 mg once daily (patients <4 years and weights <20 kg).^12^ The up titration was done in 0.5–1 mg/2–4 weeks. End doses ranged 1–8 mg/day according to the clinical need and PER plasma concentrations testing.

### PER plasma concentrations

PER plasma concentrations were measured in 79 patients using ultra‐performance liquid chromatography. We calculated the concentration‐to‐dose (CD) ratio (ng/ml per mg/kg) to adjust for the body weight. The data were compared between those with versus without enzyme‐inducing anti‐seizure medications (EIASMs). EIASMs included carbamazepine, oxcarbazepine, phenytoin, phenobarbital, and topiramate.

### Treatment outcome

We evaluated treatment outcomes and tolerability at the last outpatient clinic or through telephone follow‐up because of the COVID‐19 pandemic. We followed the efficacy and tolerability at 6 and 12 months after PER treatment. The responder was defined as ≥50% reduction in seizure frequency. Reasons for drug withdrawal were recorded by the family's reports. For the gene‐specific response to PER, we categorized genes according to the protein function.^14^ According to the result of gene‐specific response to PER, we conducted the comparison between patients with pathogenic or likely pathogenic variants of *SCN1A* and other sodium ion channels genes, *SCN1A* and other ion channel genes, *SCN1A* and other genes.

### Statistical analysis

Statistical analyses were performed with SPSS Version 26 (IBM). Baseline characteristics were given by descriptive statistics. The continuous data were presented as medians, and categorical data were presented as numbers (%). We used the Mann–Whitney U‐test to compare subgroups and continuous variables and the Pearson's chi‐square test or Fisher's exact test for categorical variables. Differences were defined as significant at *P* < 0.05. We performed multivariate logistic regression to analyze factors influencing the response rate of perampanel treatment (*P* < 0.1).

## Results

### Patients' demography

**Figure 1:**
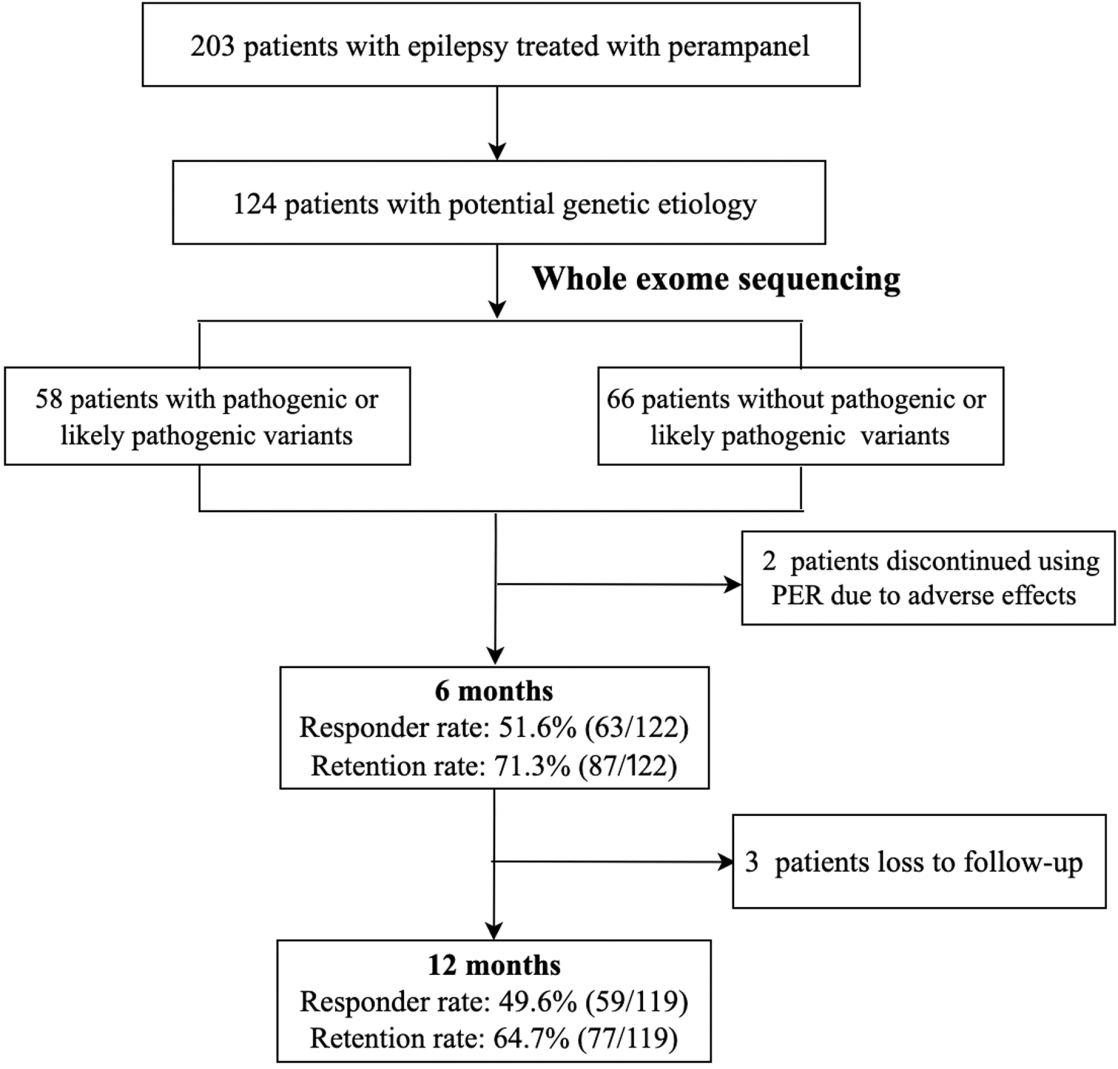
Flowchart of the patients collection and primary reason for discontinuation during follow‐up period.

A total of 124 patients, who were classified as potential genetic etiology and performed WES, were included (Fig. 1). Among them, 58 (46.8%, 58/124) had a pathogenic or likely pathogenic variant, according to ACMG guidelines. Seventy‐three (75.0%, 73/124) were diagnosed with drug‐resistant epilepsy. Seventy‐seven had developmental delay, including 11 with infantile spasms, 11 with Dravet syndrome, and five with Ohtahara syndrome. In our groups, more than half of the patients had focal epilepsy (76/124, 61.3%), including focal seizure with awareness (*n* = 9), focal seizure without awareness (*n* = 63), and focal to bilateral tonic–clonic seizures (*n* = 4). Forty‐eight patients (48/124, 38.7%) had generalized seizures, including 15 tonic–clonic seizures, nine myoclonic seizures, six epileptic spasms, six tonic seizures, and two absence seizures. On PER administration, it was often combined mostly with valproate (*n* = 53), followed by levetiracetam (*n* = 37), and lamotrigine (*n* = 16).

### Efficacy and tolerability

At 6 months' follow‐up, two patients discontinued PER because of adverse events despite it being effective. The overall response rate was 51.6% (63/122), and the seizure freedom rate was 24.6% (30/122). Thirty‐five (35/122, 28.7%) patients discontinued PER due to lack of efficacy. At 12 months, three patients were further lost to follow‐up. The overall response rate was 49.6% (59/119), and the seizure freedom rate was 26.1% (31/119). Seven additional patients discontinued PER due to a lack of efficacy during 6–12 months after PER administration. The retention rates were 71.3% (87/122) at 6 months and 64.7% (77/119) at 12 months.

**Figure 2:**
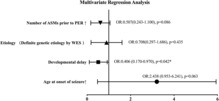
Multiple regression analysis. The variables from Table 1 with *P* < 0.1 were entered into the multivariate logistic regression analysis. *P < 0.05.

Patients' clinical profiles and PER treatment responses are shown in Table 1. No significant differences were found between the responder and non‐responder groups in terms of gender, fever sensitivity, seizure type, and presence of epileptogenic lesions in MRI scans. However, the responder group showed a higher seizure onset age, absence of developmental delay, unknown WES results, and a less number of ASMs before PER administration. On multivariate logistic regression analysis, only the developmental delay (odds ratio = 0.406, 95% CI = 0.170–0.970, *P* = 0.042) showed negative correlation with the treatment response (Fig. 2).

**Table 1:** Clinical characteristic of patients associated with perampanel response (*n* = 119).

	Total *n* = 119 (%)	Responder *n* = 59 (%)	Non‐responder *n* = 60 (%)	*P* value
Gender				0.525
Female	49 (41.18)	26 (43.33)	23 (38.98)	
Male	70 (58.82)	34 (56.67)	36 (61.02)	
Age at onset of seizure, years, median (range)	2.1 (0–13.5)	4.0 (0.6–13.5)	1.10 (0–13)	0.001*
Age at PER initiation, years, median (range)	5.0 (0.5–18)	6.0 (0.5–18.0)	5.0 (0.6–15.0)	0.840
Epilepsy duration, years, median (range)	2.0 (0–14.6)	2.0 (0–12.0)	2.5 (0–14.6)	0.234
Seizure onset age				0.000*
0–1 years	41 (34.45)	11 (18.64)	30 (50.00)	
>1 years	78 (65.55)	48 (81.36)	30 (50.00)	
Developmental delay	75 (63.03)	28 (47.46)	47 (78.33)	0.000*
Fever sensitivity	24 (20.17)	14 (23.73)	10 (16.67)	0.337
Most common seizure type				0.156
Focal	71 (59.66)	39 (66.10)	32 (53.33)	
Generalized	48 (40.34)	20 (33.90)	28 (46.67)	
Neuroimaging				0.257
Abnormal and etiologically relevant	72 (61.02)	39 (66.10)	33 (55.93)	
Normal/incidental/equivocal	46 (38.98)	20 (33.90)	26 (44.07)	
Number of ASMs prior to PER				0.002*
1	11 (9.24)	11 (18.64)	0 (0.00)	
2	18 (15.13)	9 (15.25)	9 (15.00)	
3–10	90 (75.63)	39 (66.10)	51 (85.00)	
Combination with EIASMs				0.234
With EIASMs	61 (51.26)	27 (45.76)	34 (56.67)	
Without EIASMs	58 (48.74)	32 (54.24)	26 (43.33)	
WES results				0.017*
Definite genetic etiology	58 (48.74)	22 (37.29)	36 (60.00)	
Unknown etiology	61 (51.26)	37 (62.71)	24 (40.00)	

* *P* < 0.05.

Adverse events were reported in 18.5% of patients (23 of 124 patients), including emotional problems (*n* = 8), dizziness (*n* = 5), fatigue (*n* = 3), ataxia (*n* = 3), enuresis (*n* = 2), and weight gain (*n* = 2). The mean CD ratio (ng/mL per mg/kg) did not show a statistical difference between patients with adverse events and those without adverse events.

### Perampanel plasma concentrations

**Figure 3:**
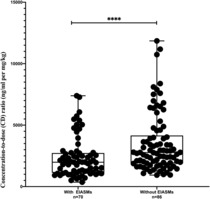
Serum concentrations of perampanel. Comparison of patients by administration of enzyme‐inducing anti‐seizure medicines. **** indicates *P* value < 0.0001.

PER plasma concentrations were measured for 156 times in 79 patients administering a median perampanel dose of 2 (1–8) mg. The PER plasma concentration was 334.2 ± 218.3 ng/mL (range, 59.90–1259.41 ng/mL), with a CD ratio of 2984.8 ± 2166.1 ng/mL per mg/kg. The CD ratio was significantly different between patients with and without EIASMs. We observed a lower mean CD ratio in patients on EIASMs than in those not on EIASMs (2329.2 ± 1626.2 vs. 3518.5 ± 2401.5, *P* = 0.000, Fig. 3). No significant differences in the mean CD ratio were found in male versus female (3304.7 ± 2479.1 vs. 2537.0 ± 1539.8, *P* = 0.174), in PER responders versus non‐responders (3121.8 ± 2190.5 vs. 2894.2 ± 2252.0, *P* = 0.515), or in those with versus without adverse effects (3269.1 ± 2352.3 vs. 2890.2 ± 2116.7, *P* = 0.282).

### Gene‐specific response to perampanel

Through WES, 58 patients with pathogenic or likely pathogenic variants in 27 genes were determined to have identified genetic etiology. Their clinical features are shown in Table S1. Response rates for these 27 different genes according to gene enrichment analysis were valued by sorting them into different gene sets according to protein function.^14^

**Figure 4:**
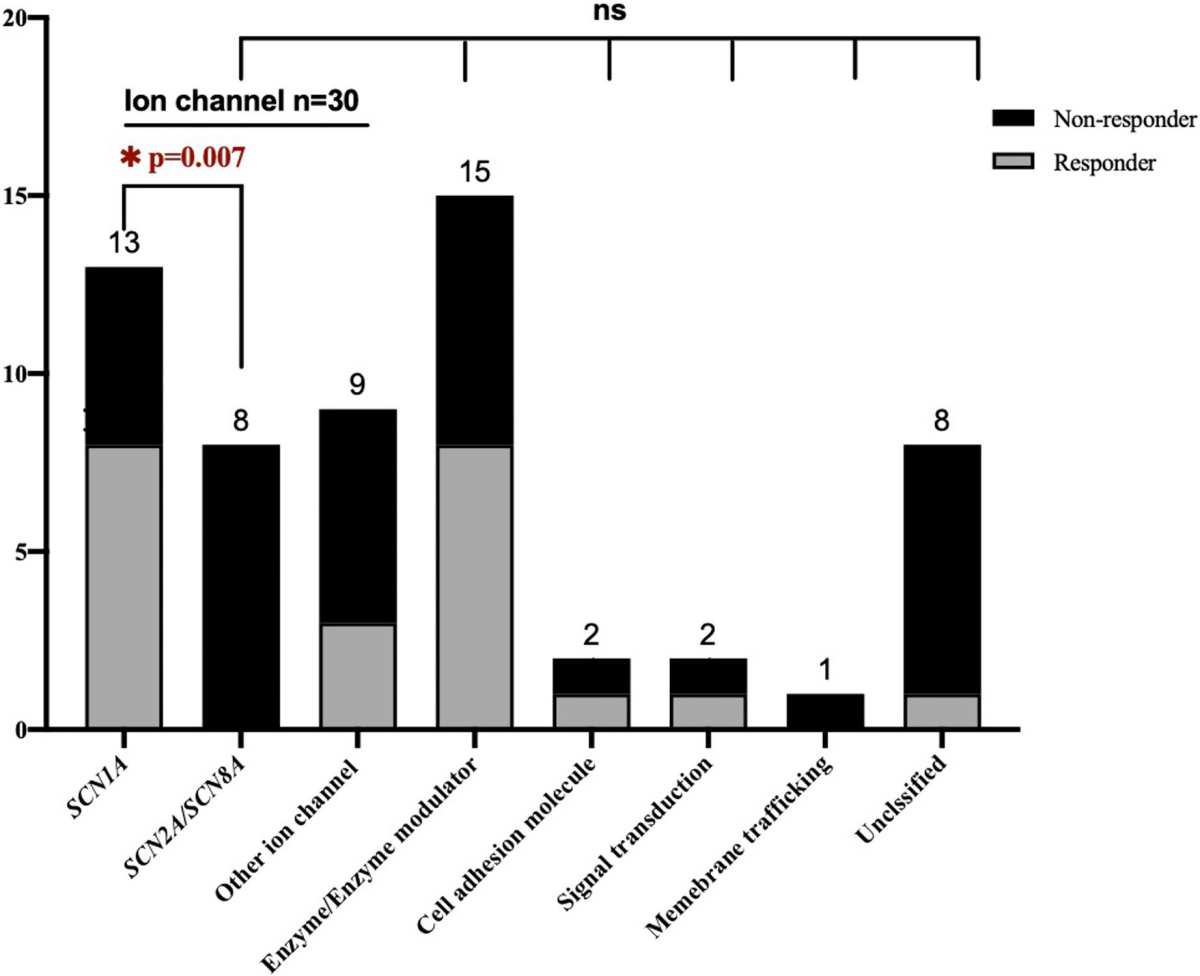
Gene‐specific responses to PER. The response rates for these detecting genes from our patients were valued by sorting them into different gene sets according to protein function (Table S2). There were no statistically differences among these groups. By dividing the group with the largest patients‐the ion channel into 3 subgroups, it showed patients with the *SCN1A* gene were more likely to respond to PER treatment than those with other sodium channel genes. *P < 0.05.

They were divided into six groups: ion channel, enzyme/enzyme modulator, cell adhesion molecule, signal transduction, membrane trafficking, and unclassified (Table S2). However, there were no statistical differences among these groups (Fig. 4, *P* = 0.420).

We further divided the ion channel group into three subgroups: Nav1.1 (*SCN1A*), other sodium channels (*SCN2A*, *SCN8A*), and other ion channels. Interestingly, the response rates were different between the *SCN1A* gene and other sodium channel genes (*SCN2A* and *SCN8A*). Patients with the *SCN1A* gene were more likely to respond to PER treatment than those with other ion channel genes (*P* = 0.018), and those with other sodium channel genes (Fig. 4, *P* = 0.007), and the clinical features in these two groups did not differ significantly (Table S3). We then compared the responder rate between 13 patients with *SCN1A* gene and 45 patients with other genes, and also showed statistical difference (*P* = 0.042). Patients with variants in the SCN1A gene displayed marked efficacy. Furthermore, among the 58 patients with positive results of WES, the multivariate logistic regression analysis showed that carriers with variants in the SCN1A gene had a better response compared to those with variants in other genes (OR = 7.124, 95% CI = 1.306–38.860, *P* = 0.023).

The enzyme/enzyme modulator group was also divided into two subgroups: enzyme and enzyme modulator. No significant between‐subgroup difference was found, but patients in the enzyme subgroup, including those with genes, such as *GNAO1*, *CHD2*, and *PAFAH1B1*, showed a good response rate (4/5, 80%).

## Discussion

Our real‐world study demonstrated that PER is well‐tolerated and effective in children with known and presumed genetic epilepsy. The response rate and seizure freedom rate reached 49.6% and 26.1% at 12 months, respectively, which is comparable with that reported in other pediatric populations, and adverse events were lower when compared to previous studies.^10^, ^15^, ^16^

The positive rate by WES in our group was nearly 50%, which was higher than those in previous studies.^17^ The reason was that patients were selected strictly according to our inclusion and exclusion criteria. Benefiting from the high positive rate, we obtained a relatively large sample of patients for further gene‐specific analysis. A variable degree of intellectual disability and behavioral comorbidities are commonplace in children with epilepsy,^2^ especially in developmental and epileptic encephalopathy (DEE), which is mainly related to genetic etiology. Using ASMs without significant effects on child neurodevelopment is important. Long‐term effects of PER on cognition in adolescents have been studied, indicating that PER did not have significant effects on cognitive parameters except for attention.^18^ PER is a suitable choice for pediatric epilepsy. Our findings show that developmental delay was strongly associated with worse treatment response. A more severe genetic insult is commonly found in children with more profound developmental delay and drug‐resistant seizures. Few studies have evaluated PER's efficacy according to an intellectual/developmental status. A small study showed the response rate of PER was 62.5% among patients with borderline‐to‐normal developmental status and 50% among those with mild‐to‐severe delays; however, no between‐group differences were found due to the very small sample size.^19^

Interestingly, we found that patients with the *SCN1A* gene had good outcomes after PER treatment more often than patients with other sodium channel genes (including *SCN1A*, *SCN2A* and *SCN8A*), and clinical features, such as the age of first seizure onset and developmental delays, had no between‐group statistical significance. For further studies, we found that carriers with variants in the *SCN1A* gene showed a better response compared to patients with other genes discovered in our group. *SCN1A* gene variation is a main cause of Dravet syndrome. In a large retrospective survey of 574 patients with Dravet syndrome, only 9.4% were seizure free in the previous 3 months.^20^ In our cohort 11 out of 13, *SCN1A*‐related patients were diagnosed with Dravet syndrome, and 63.6% (7/11) of them responded to PER treatment. Previous case series studies displayed a similar response rate.^10^, ^13^, ^21^, ^22^ A recent study in epilepsy associated with a *SCN1A* pathogenetic variant (responder rate: 11 of 17, 64.7%) was in line with our findings.^22^ We then reviewed the published variants in *SCN1A* gene with PER treatment, but no discrepant distribution of effective and ineffective variants were found (sup Fig. 1). Exciting results of PER in *SCN1A*‐gene‐related Dravet syndrome have been explained by reduction of GABAergic inhibitory interneurons arising from the AMPA receptor‐mediated excitotoxic death.^10^ PER may be a proper option for *SCN1A*‐related Dravet syndrome. In contrast, a poor response rate of PER was found for those with other voltage‐gated sodium channel genes (*SCN2A* and *SCN8A*). The expression location of different types of neurons and the functional change of variants between the *SCN1A* gene and other voltage‐gated sodium channel genes are noted discrepancies. Different effects of different sodium channels on PER might be related to their discrepancies in distribution and functional changes.^23^, ^24^, ^25^ Interestingly, a deep investigation would be a further step in PER precise treatment of genetic epilepsy.

PER partially suppressed seizures and involuntary movements in two patients with early‐onset epileptic encephalopathy caused by the *GNAO1* gene, reached a 100% seizure reduction. Interestingly, Nissenkorn et al^22^ reported the same finding (responder rate: 4 of 4, 100%) for those with pathogenic variants in the *GNAO1* gene. Including ours, there have been six reported cases. The similar findings warrant further investigation for this rare type of DEE.

The *GNAO1* gene encodes the alpha subunit of the heterotrimeric guanine nucleotide‐binding proteins (Gαo), and variants in *GNAO1* gene have been demonstrated with two main phenotypes: Epileptic encephalopathy, early infantile, 17 (EIEE 17, OMIM 615473) and Neurodevelopmental disorder with involuntary movements (NEDIM, OMIM 617493). These two phenotypes showed limited response to pharmacological treatments and had poor outcome with severe development delay.^26^ It has been demonstrated that *GNAO1* pathogenic variants associated with epilepsy result in a loss‐of‐function biochemical behavior related to the control of cAMP levels.^27^ Meanwhile, AMPA‐receptor antagonists, such as perampanel, have been shown to restore up‐regulation of cAMP response element‐binding protein (CREB) phosphorylation.^28^ PER application in *GNAO1*‐related epilepsy has not been studied before; it needs large samples and further mechanistic research.

Naturally, we should not ignore that PER clearance can be markedly increased by the CYP3A4 enzyme inducers like carbamazepine, oxcarbazepine, phenytoin, phenobarbital, and topiramate (EIASMs).^29^ EIASMs led to lower PER plasma concentrations in our study, and a higher dosage of PER was required. On the contrary, when reducing or withdrawing EIASMs, plasma PER concentrations are likely to increase, which might bring about potential adverse effects. Therefore, plasma concentrations must be monitored when using or withdrawing EIASMs. The average plasma concentration in our study tended to be higher than the plasma concentration previously reported in a Japanese trial, but the average CD ratio did not differ significantly.^30^ There is no discrepancy between the PER plasma concentrations of the responder and non‐responder groups.

There were several limitations in our study. This was an uncontrolled, individual observational study. Despite having the largest reported sample to date, the heterogeneity of epilepsy phenotypes and genotypes lead to a larger number of patients demonstrating a responder difference among diverse genes. It was also a retrospective study, which lacked some details of medical records such as the accurate seizure frequency of each patient, which limited the ability to test a priori hypotheses further. Prolonged follow‐up and larger samples are needed to determine the gene‐specific response to PER.

## Conclusions

In conclusion, this long‐time real‐world study supports the use of PER in treatment of children with known and presumed genetic epilepsy. Further analysis based on gene‐specific epilepsy demonstrated a valuable direction for the precision treatment of PER. Therefore, a definite genetic diagnosis is important in PER usage. For more‐profound research, larger samples and longer follow‐up periods for each group of genes are required.

## Author Contributions

Pu Miao, Jianhua Feng, Shuang Wang contributed to the conception of the study; Yanfang Li performed the experiment; Pu Miao, Shuang Wang, Qunyan Su contributed significantly to analysis and manuscript preparation; Pu Miao, Xueying Zhu, Ye Wang performed the data analyses and wrote the manuscript; Wenqin Jin, Lingyan Yu, Sha Xu helped perform the analysis with constructive discussions.

## Funding Information

The project is supported by the grants from the National Natural Science Foundation of China 82101515 and Public Projects of Zhejiang province (Grant Number: 2021C03104).

## Conflict of Interest

The authors declare that there is no conflict of interest.

## Declarations

Ethics approval and consent to participate. The study was approved by the local ethics committee (2021‐0605/IR2021398), and the informed consent to participate was obtained from participants.

## Consent for Publication

Not applicable.

## Supporting information


**Figure S1.** Representation of published and our cohort's variants treatment response across the *SCN1A* protein. The alpha subunit consists of four homologous domains (D1–4) each formed of six transmembrane segments (S1–S6). Segment 4 represents the voltage sensor and segments S5–6 the pore region. Red rhombus denotes effective variants by PER, blue triangle denotes non‐effective variants by PER.

**Table S1.** The phenotype and genotype of patients with positive WES results.
**Table S2.** Gene classification according to protein function.
**Table S3.** The clinical features in these two groups.

## Data Availability

Not applicable.
