# Supplementary figures and images for: Efficacy of perampanel in pediatric epilepsy with known and presumed genetic etiology
Source: Ann Clin Transl Neurol. 2023 Jun 16;10(8):1374–82. doi: 10.1002/acn3.51828 (PMC10424658; doi:10.1002/acn3.51828)

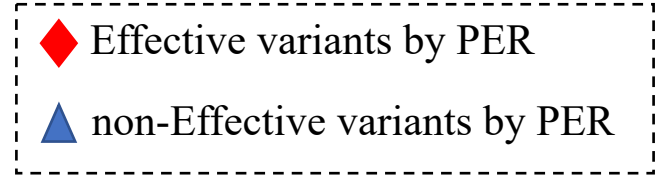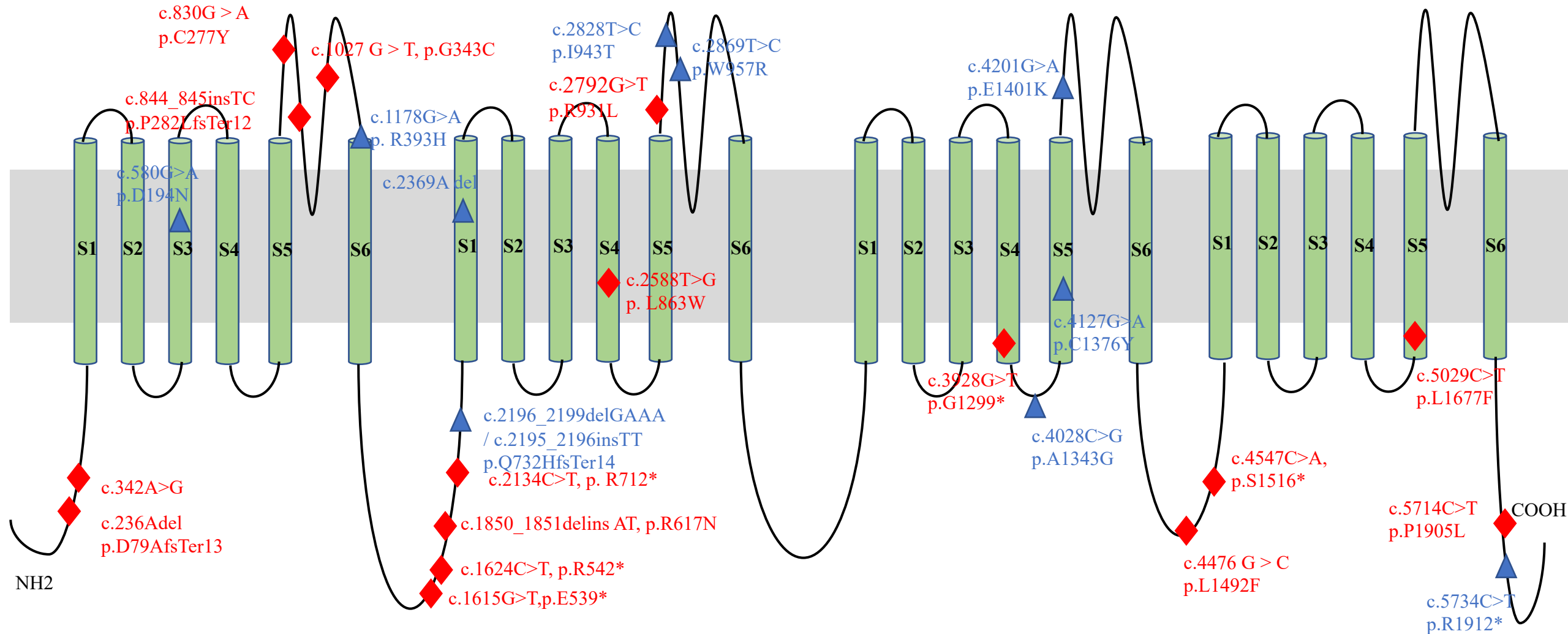

Supplement: Supplementary file 1 — Figure S1. Representation of published and our cohort's variants treatment response across the SCN1A protein. The alpha subunit consists of four homologous domains (D1–4) each formed of six transmembrane segments (S1–S6). Segment 4 represents the voltage sensor and segments S5–6 the pore region. Red rhombus denotes effective variants by PER, blue triangle denotes non‐effective variants by PER. [file ACN3-10-1374-s001.pdf]
