# Supplementary material for: Efficacy of perampanel in pediatric epilepsy with known and presumed genetic etiology
Source: Ann Clin Transl Neurol. 2023 Jun 16;10(8):1374–82. doi: 10.1002/acn3.51828 (PMC10424658; doi:10.1002/acn3.51828)
Supplement: Supplementary file 2 — Table S1. The phenotype and genotype of patients with positive WES results. Table S2. Gene classification according to protein function. Table S3. The clinical features in these two groups. [file ACN3-10-1374-s002.docx]

**Supplement table 1 The phenotype and genotype of patients with positive WES results**

| **Gender** | **Age(year)** | **Age at seizure onset(year)** | **Developmental delay** | **Variants** | **Current ASMs** | **Responder** | **Seizure-free** |
| --- | --- | --- | --- | --- | --- | --- | --- |
| F | 8 | 2 | Y | ADGRV1 c.12211C>T p.R4071X | LEV | Y | Y |
| F | 12 | 12 | N | ADGRV1 c.443C>A p.S148X | NZP,LEV | N | N |
| M | 0.7 | 0.4 | Y | ATP7A c.2916+1G>A splice | PB,OXC | N | N |
| F | 1.1 | 0 | Y | CACNA1E c.1054G>A p.G352R | VPA,LGT | N | N |
| F | 6 | 5.5 | Y | CHD2 c.2095C>T p.R699W | LEV，VPA | N | N |
| F | 3 | 3 | Y | CHD2 loss1(EXON:2-38) | LGT,NZP | Y | N |
| F | 18 | 8 | N | CSTB c.116_117delAG p.K39SfsTer*37 | LEV,VPA | Y | N |
| M | 10 | 4 | Y | DEPDC5 c.1777C>T p.P593S | CBZ | Y | Y |
| F | 12 | 0.7 | Y | EFHC1 c.1492+1G>T splice | OXC,LGT | N | N |
| F | 3 | 0.5 | N | FGF12 c.334G>A p.G112S | OXC, LEV | Y | Y |
| F | 3 | 0.1 | Y | GABRA1 c.1079C>A p.P360H | OXC, CLB | N | N |
| F | 1 | 2 | Y | GNAO1 c.143C>T, p.T48I | TPM,NZP,VPA | Y | N |
| M | 0.5 | 0 | Y | GNAO1 c.118G>C p.G40R | TPM,CZP,LEV | Y | N |
| M | 10 | 5 | Y | GRIN2A c.1321A>T p.K441X | VPA | N | N |
| F | 8 | 4 | N | GRIN2A c.3695C>G p.S1232C | OXC,LEV | Y | N |
| M | 1.8 | 0.6 | Y | IRF2BPL c.1252A>G p.K418E | LEV,TPM | N | N |
| F | 5 | 1.5 | Y | KCNB1 c.990G>T,p.Glu330Asp | TPM,NZP | N | N |
| M | 8 | 1.5 | Y | KCNQ2 c.1461G>C p.E487D | VPA,LGT,TPM,CLB | N | N |
| M | 1.4 | 1.3 | Y | KCNT1 c.1183G>A P.R398Q | VPA,CBZ | N | N |
| M | 4 | 2.5 | N | KCNT1 c.2893G>A p.A965 | VPA | Y | N |
| M | 8 | 5 | Y | KCNT1 p.Ala934Thr | TPM,NZP | N | N |

| **Gender** | **Age(year)** | **Age at seizure onset(year)** | **Developmental delay** | **Variants** | **Current ASMs** | **Responder** | **Seizure-free** |
| --- | --- | --- | --- | --- | --- | --- | --- |
| M | 6 | 4 | N | NF1c.7563_7672delCATGAGCCTGp.s2521fsTer24 | VPA,LGT,CLB | N | N |
| M | 10 | *9* | N | NPRL3 loss1(EXON:3-6) | OXC,VPA | N | N |
| M | 4 | 3 | Y | PAFAH1B1 c.1002+5G>A splice | VPA,TPM | Y | Y |
| M | 4 | 3 | Y | PAFAH1B1 c.1002+5G>A splice | VPA,TPM | Y | Y |
| F | 2 | 1 | Y | PCDH19 c.918C＞G p.Y306X | TPM,CZP，LEV | Y | Y |
| F | 9 | 8 | Y | PCDH19 c.C15555T p.R519X | VPA,LEV,TPM | N | N |
| M | 0.5 | 0.4 | Y | SCN1A c.1615G>T p.E539X | TPM | Y | N |
| M | 5 | 0.3 | Y | SCN1A c.1850_1851del,p.Arg617ThrfsTer10 | TPM,VAP,NZP | Y | N |
| M | 7 | 8 | N | SCN1A c.2134C>T,p.Arg712X | LEV,VPA | Y | Y |
| F | 6 | 0.5 | Y | SCN1A c.2196_2199delGAAA / c.2195_2196insTT | TPM,LEV | N | N |
| M | 2.3 | 0.7 | Y | SCN1A c.2792G>T p.R931L | VPA,ZNS | Y | N |
| F | 9 | 0.8 | Y | SCN1A c.2828T>C p.I943S | LEV | N | N |
| M | 4 | 0.3 | Y | SCN1A c.3928G>T p.G1310X | TPM, CLB,VPA | Y | N |
| M | 3 | 0.1 | Y | SCN1A c.4028C>G p.A1343G | TPM.VPA | N | N |
| F | 2 | 1 | Y | SCN1A c.4127G>A p.C1376Y | LEV | N | N |
| F | 3 | 1 | N | SCN1A c.5714C>T p.P1905L | TPM, LEV，VPA | Y | N |
| F | 5 | 0.3 | Y | SCN1A c.830G>A 3 p.C277Y | VPA,LEV | Y | N |
| M | 6 | 0.3 | N | SCN1A c.844_845insTC p.P282LfsTer12 | LEV,TPM | Y | N |

| **Gender** | **Age(year)** | **Age at seizure onset(year)** | **Developmental delay** | **Variants** | **Current ASMs** | **Responder** | **Seizure-free** |
| --- | --- | --- | --- | --- | --- | --- | --- |
| M | 2 | 0.4 | Y | SCN1A，c.580G>A,p.D194N | LEV | N | N |
| M | 2 | 0.3 | Y | SCN2A c.2815C>G p.L939V | VGB,TPM | N | N |
| F | 1.2 | 0 | Y | SCN2A c.2995G>A, p.E999K | OXC,NZP | N | N |
| F | 2 | 0.7 | Y | SCN2A c.3531G>A,p.E1211K | TPM,VPA,VGB | N | N |
| M | 2 | 0 | Y | SCN2A c.4972C>T p.P1658S | CLB，CBD，TPM | N | N |
| M | 1.7 | 0 | Y | SCN2A c.640T>C p.S214P | VGB,TPM | N | N |
| M | 5 | 5 | N | SCN8A c.1588C>T p.R530W | OXC,VPA | N | N |
| F | 3 | 0.4 | Y | SCN8A c.3968C>G p.A1323G | CBZ, VPA, OXC | N | N |
| F | 3 | 0 | Y | STXBP1 c.798T>G p.Y266* | TPM,VPA,LEV | N | N |
| M | 3 | 0.1 | Y | STXBP1 c.902+5G>A(IVS10) | LEV, TPM, LGT | N | N |
| F | 3 | 2.1 | Y | SYNGAP1 c.1898T>C p.L633P | VPA | N | N |
| M | 2 | 0.3 | Y | TBC1D24 c.229_c.240 delATCGTGGGCAG,P.I77_K80del;c.116C>T,p.A39V | CLB | N | N |
| M | 7 | 3 | N | TSC1 c.272C>A p.S91X | TPM,OXC | Y | Y |
| F | 8 | 8 | N | TSC1 c.663+1G>T splic | OXC | N | N |
| M | 5 | 2 | Y | TSC2 c.2866C>T p.R956 | VPA,LGT | N | N |
| M | 7 | 4 | Y | TSC2 c.3070G>A p.E1024K | VPA,NZP | N | N |
| M | 14 | 8 | Y | TSC2 c.3193A>G p.K1065E | NZP,TPM,CBZ,LCM | Y | Y |
| F | 4 | 0 | Y | TSC2 c.4787G>A p.G1596D | VPA,LEV | N | N |
| M | 4 | 2 | N | TSC2  c.1258-2A>G splice | / | Y | N |

LTG Lamotriaine , ZNS Zonisamide, CBZ Carbamazepine, OXC Oxcarbazepine, LCM Lacosamide , NZP Nitrazepam， CZP Clonazepam， VGB Vigabatrin, LEV Levetiracetam, VPA Valproic acid, LCM Lacosamide, TPM Topira

**Supplement table 2 Gene classification according to protein function**

| **Gene** | **Ion channel** | **Enzyme/enzyme modulator** | **Cell adhesion molecule** | **Signal transduction/** | **Membrane trafficking** | **Unclassified** |
| --- | --- | --- | --- | --- | --- | --- |
| ADGRV1 |  |  |  |  |  | √ |
| ATP7A |  |  |  |  |  | √ |
| CACNA1E | √ |  |  |  |  |  |
| CHD2 |  | √ |  |  |  |  |
| CSTB |  | √ |  |  |  |  |
| DEPDC5 |  |  |  |  |  | √ |
| EFHC1 |  |  |  | √ |  |  |
| FGF12 |  |  |  | √ |  |  |
| GABRA1 | √ |  |  |  |  |  |
| GNAO1 |  | √ |  |  |  |  |
| GRIN2A | √ |  |  |  |  |  |
| IRF2BPL |  |  |  |  |  | √ |
| KCNB1 | √ |  |  |  |  |  |
| KCNQ2 | √ |  |  |  |  |  |
| KCNT1 | √ |  |  |  |  |  |
| NF1 |  |  |  |  |  | √ |
| NPRL3 |  | √ |  |  |  |  |
| PAFAH1B1 |  | √ |  |  |  |  |
| PCDH19 |  |  | √ |  |  |  |
| SCN1A | √ |  |  |  |  |  |
| SCN2A | √ |  |  |  |  |  |
| SCN8A | √ |  |  |  |  |  |
| STXBP1 |  |  |  |  | √ |  |
| SYNGAP1 |  |  |  |  |  | √ |
| TBC1D24 |  | √ |  |  |  |  |
| TSC1 |  | √ |  |  |  |  |
| TSC2 |  | √ |  |  |  |  |

**Supplement table 3 The clinical features in these two groups**

**(SCN1A vs *SCN2A/SCN8A*)**

| **Clinical features** | **Total  n=21 (%)** | **SCN1A n=13 (%)** | **SCN2A,SCN8A**  **n=8 (%)** | **P value** |
| --- | --- | --- | --- | --- |
| **Gender, female** | 8 (38.1%) | 5 (38.5%) | 3 (37.5%) | 1.000 |
| **Age at onset of seizure** |  |  |  | 1.000 |
| 0-1 y | 19 (90.9%) | 12 (92.3%) | 7 (87.5%) |  |
| >1y | 2(9.1%) | 1(7.7%) | 1(12.5%) |  |
| **Developmental delay** | 15 (71.4%) | 9 (69.2%) | 6 (75.0%) | 1.000 |
| **Number of ASMs prior to PER** |  |  |  | 0.240 |
| 1 | 0(0%) | 0(0%) | 0(0%) |  |
| 2 | 3(14.3%) | 3(23.1%) | 0(0%) |  |
| 3-7 | 18(85.7 %) | 10(76.9%) | 8(100%) |  |
| 8-10 | 0(0%) | 0 (0.0%) | 0 (0.0%) |  |
